# Recognition of Gonadal Development in *Eriocheir sinensis* Based on the Impulse of Love at First Sight

**DOI:** 10.3389/fphys.2022.793699

**Published:** 2022-04-27

**Authors:** Jingjing Jiang, Shengyan Su, Ting Lai, Wenrong Feng, Feifan Li, Can Tian, Yang Gao, Brian Peelekelo Munganga, Yongkai Tang, Pao Xu

**Affiliations:** ^1^ Wuxi Fisheries College, Nanjing Agricultural University, Wuxi, China; ^2^ Key Laboratory of Genetic Breeding and Aquaculture Biology of Freshwater Fishes, Ministry of Agriculture, Freshwater Fisheries Research Center, Chinese Academy of Fishery Sciences, Wuxi, China; ^3^ National Demonstration Center for Expermental Fisherise Science Education, Shanghai Ocean University, Shanghai, China

**Keywords:** *Eriocheir sinensis*, ovarian development, pheromone, sex motivation, reproduction

## Abstract

Given the difficulty in identifying individuals with different degrees of ovarian development, we developed a new device utilizing the hypothesis of mutual attraction behavior between male and female crabs with mature gonads by releasing the sexual pheromone so they could be examined. From a total of 40 female crabs, 10 were isolated within half an hour. Histological analysis showed that the ovaries of crabs in the isolated group were in stage IV, while those of the control groups were in stage III. In addition, progesterone (PROG) in experimental groups was significantly reduced compared with the control group (*p* < 0.05), but no significant difference was detected in estradiol (E2). In response to the different developmental stages, hemolymph biochemical indices and the determination of gonadal fatty acids profiles were explored. The results indicated only C18:4 showed a significant difference between these two groups. A transcriptome was generated to determine the genes involved in the mutual attraction process; differentially expressed genes (DEGs) were significantly related to gonadal development. Therefore, the device can be used to isolate Chinese mitten crabs with stage IV ovarian development.

## Introduction

“Fall in love at first sight” is an aesthetic idea that suggests the occurrence of strong attraction between two parties at first sight (Lu et al., 2020). As a saying goes, beauty is in the eye of the beholder, and they attract and approach each other constantly (Harrison & Shortall, 2011). This is due to the sexual pheromones secreted by men and women with mature gonads, which are transmitted through sensory organs to the nerve center to induce sexual motivation (Grammer et al., 2005), whose behavioral manifestations are closed to each other. Pheromones are substances released into the environment by animals and can trigger specific chemical reactions among different individuals of the same species, which is of great significance to maintaining the reproduction of a population (Wyatt, 2010). Crustaceans also generate different pheromones in addition to their own growth- and reproduction-related hormones, attracting the opposite sex during the breeding season (Shimomura et al., 2010), which is similar to the idea of falling in love at first sight in humans.

The Chinese mitten crab (*Eriocheir sinensis*), commonly known as the river crab, is the main aquaculture species of important economic value in China. Given the special flavors and high nutritional value of their gonads, female Chinese mitten crabs are popular with consumers (Shao et al., 2013; Wang et al., 2016). The status of gonadal development in Chinese mitten crabs directly impacts the overall perceived edible quality of the final product and market price (Shao et al., 2014). Morphological characteristics are the most intuitive way to evaluate the growing status of the Chinese mitten crab, where high value often indicates their reproductive performance (Barry, 2010), but the relationship between ovarian development state and morphology has not been definitively concluded. We have no way to intuitively judge the status of ovarian development by the specifications of crabs. Due to the cost and error involved in the manual selection of crabs, how we estimate the status of gonadal development accurately has become an urgent problem to be solved.

Hormones have also played a key role in ovarian development through progressive activation of the hypothalamic-pituitary-gonadal axis (Wierman, 2007; Ye et al., 2008). Estradiol and progesterone play a key role in the expression of receptive lordosis behavior in the nervous system in female mice, which contribution to female motivation to seek out male pheromones requires further study (McCarthy et al., 2018). However, studies have shown that estradiol, perhaps with the help of progesterone, was asked to trigger a preference for the male pheromone, and they are potent sexual communication signals that induce attractive behavior in potential mates in brown mice and house mice (Varner et al., 2018). Hence, estradiol and progesterone act as sex pheromones to a certain extent, which indicates that sex pheromones can indirectly reflect the ovarian developmental state. In many animal species, mature individuals that are ready to mate release chemical signals (sex pheromones) that are detected and used by other individuals to identify potential mates (Yambe et al., 1999). Pheromones are randomly released into the water through urine. In crabs, signal delivery is often aided by self-generated fanning currents that flush chemicals towards receivers, which themselves might actively pull water towards their sensory structures (Thiel & Breithaupt, 2011). The pheromone is detected by crabs using specific chemical sensors (aesthetasc sensilla) on the antennules (Kamio et al., 2005). This signal may act in a short time to stimulate courtship behavior in the opposite sex (Kamio et al., 2014). Mature individuals are attracted to water scented with substances originating from the opposite sex of the same species about a meter or so away. Previous investigations have verified that the crabs of the opposite sex will generate sexual motivation *via* pheromones (Herborg et al., 2006; Wyatt, 2015) and show varying sensitivity to pheromones at different stages of reproduction. Therefore, we hypothesized that the status of gonadal development can be estimated by this behavior.

Based primarily on the above theory, we designed a device to isolate Chinese mitten crabs with higher gonadal development, and isolated and unisolated crabs were collected by this device to investigate differences in hemolymph biochemical indices and reproductive performance of Chinese mitten crab with unsynchronized ovarian development. The isolation effect of the device used to identify the ovarian development degree of Chinese mitten crabs was evaluated, and the metabolic regulation mechanism and related gene expression differences of the non-synchronous development of ovaries in the Chinese mitten crab population were revealed, which provided basic data and a reference basis for the breeding of the Chinese mitten crab.

## Materials and Methods

### Crab Management

A total of 40 female and 20 male randomly sized Chinese mitten crabs with complete appendages and good vitality were selected from Jiangsu Haorun in October, 2020; they were kept for 1 week and fed with chilled fish and corn daily. Crab handling and experimental procedures were carried out in accordance with the guidelines for the care and use of animals for scientific purposes set by the Institutional Animal Care and Use Committee of the Freshwater Fisheries Research Center and approved by the animal ethics committee of the Chinese Academy of Fishery Sciences.

### Developed Device Description

**FIGURE 1:**
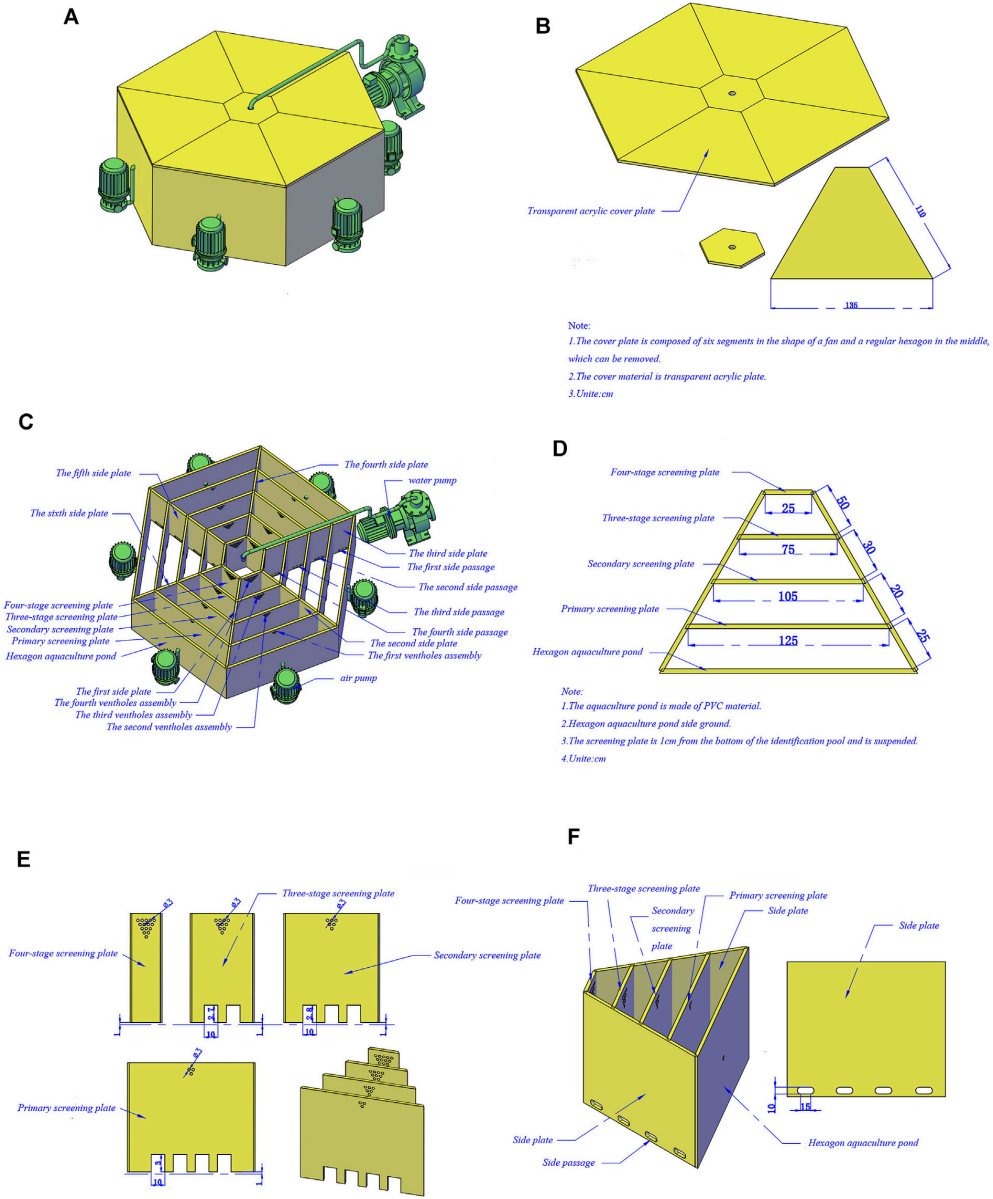
Isolation device pattern drawing of Eriocheir sinensis with different ovarian developmental statues. **(A)** Device appearance drawing; **(B)** Structure drawing of cover plate; **(C)** Internal structure drawing of the device; **(D)** Distribution graph of screening plate; **(E)** Structure drawing of screening plate; **(F)** Distribution graph of side passages.

We developed a polygon device comprising a discriminating area, a transparent cover plate located at the top of the area, and an external mechanical aerating device (Figure 1A,B). The whole device is shaped like a polygon maze consisting of four concentric loops and a central zone separated by screening plates and side plates. The center zone is the area where the male crabs gathered, and the width of four concentric loops increases following the Fibonacci sequence from the outside to the inside (Figure 1C). The screening plates are not sealed to the baseboard to facilitate easy drainage and suction, and some pheromones dissolved in water can be diffused to the periphery with gradient descent (Figure 1D). Meanwhile, the screening plates have unilateral gates that effectively prevent crabs from returning to the outer loops and air holes by which the male crab pheromone can spread from the male crab zone to the periphery (Figure 1E). Otherwise, a growing number of air holes increases the pheromone concentration from the outside to the inside, thus being more attractive to the females. Unilateral gates are distributed asymmetrically, the number of which decreases to increase cross-channel difficulty, and the side plate is fitted with side passages so crabs have to constantly traverse to find unilateral gates to get close to the opposite sex (Figure 1F).

Firstly, in total 40 female Chinese mitten crabs were distributed randomly and equally in the outmost circular track and 10 male crabs were selected in the central male crab area. Then the transparent acrylic plate was covered and the monitoring device was activated; the unilateral gate on the primary screening plate was opened. The test site was free from any interference, and the test was controlled within half an hour. Isolated tests were repeated four times. The remaining females that were not accurately isolated were re-isolated, while the males in the central area were partially replaced. Before screening female crabs, we put some female crabs in the central male crab area and male crabs in the outmost circular track. The male crabs were initially screened, and the male crabs with mature gonads were screened for the female screening experiment.

### Crab Sampling and Data Collection

Crabs were isolated as an experimental group by the designed device, and the control group was randomly chosen from the remaining crabs. The wet weight, body length, body width, and body height of the crabs were measured first, and then the hemolymph was exsanguinated from the base of the third walking limb of each crab. After incubation at 4°C for 24 h, the serum was obtained from the hemolymph by centrifugation at 4500 rpm and 4°C for 10 min and then stored at −80°C for further analysis. Gonads and hepatopancreas were dissected and weighed separately and then stored at −80°C until use. The GSI (gonadosomatic index) and HSI (hepatosomatic index) of the crabs were calculated using the following formulas:

$$\text{GSI}\left(\text{\%}\right)=\frac{\text{gonad wet weight}}{\text{body wet weight}}\text{∗}100$$

$$\text{HSI}\left(\text{\%}\right)=\frac{\text{hepatopancreas wet weight}}{\text{body wet weight}}\text{∗}100$$

### Biochemical Analysis

The hepatopancreas samples were carefully weighed to about 0.1 g and homogenized in a 900 ul 0.85% saline solution. The homogenate was centrifuged at 2500 rpm at 4°C for 10 min, and the upper lipid layer was discarded. The supernatant was carefully collected and used for amylase (AMS) activity determination. Malondialdehyde (MDA), superoxide Dismutase (SOD), acid phosphatase (ACP), alkaline phosphatase (AKP), and total antioxidant capacity (T-AOC) activities were determined in the serum. All indicators were carried out by the diagnostic reagent kits (Nanjing Jiancheng Bioengineering Institute, Nanjing, China) according to the manufacturer’s instructions. Estradiol (E2) and progesterone (PROG) determinations in hemolymphs were carried out utilizing the enzyme immunoassay kits (ELISA) (Yuanye, Biotechnology Company, Shanghai, China).

### Histological Analysis of the Ovary

The ovarian tissue samples fixed in Bouin’s solution for 24 h were further dehydrated, cleaned, and equilibrated using ethanol, toluene, and xylene, respectively, and then embedded in paraffin. After that, they were sectioned with a microtome at a thickness of 5 μm, stained with hematoxylin and eosin (HE), and then examined with a microscope.

### Determination of Gonadal Fatty Acids

The total lipid of the gonadal tissue was extracted using chloroform-methanol-H2O (2:2:1,v/v/v). The fatty acids were methylated with 1 mol/L KOH-methanol and 0.5 mol/L sulfuric acid methanol solutions and then extracted with n-heptane. Fatty acids methyl esters were measured directly by GC-MS (Long & Antoniewicz, 2014) (Agilent 7890B-5977 A).

### RNA Isolation, RNA-Seq Library Preparation, and Next-Generation Sequencing

Three sets of gonadal tissue each from the experimental group and control group were randomly selected. Total RNA was extracted from the tissues using Trizol (Invitrogen, Carlsbad, CA, United States) according to the manufacturer’s instructions, and genomic DNA was removed with DNase I (Takara). The quality of RNA was estimated by an Agilent 2100 Bioanalyzer (Agilent, Shanghai, China). Only high-quality RNA samples (OD260/280 range of 1.8–2.2, RIN≥8.0) were used to construct the sequencing library. The sequencing libraries were generated by the TruSeq RNA sample prep kit (Illumina) according to the manufacturer’s recommendation. Then, the library was sequenced using Illumina Hiseq (2000) and (100) bp single-end reads were obtained.

### Transcriptome Assembly and Annotation

Before assembling, the raw reads obtained after sequencing were filtered to remove adaptor and low-quality sequences to obtain clean reads through a fastqc filter (Biomarker Technologies, Beijing). Then, the clean reads were assembled using the Trinity software (v2.0.6) (Vasani, N. (2019). The assembled transcriptomes were annotated using NCBI, NR, KEGG, Swissprot, and KOG databases (Ashburner et al., 2000).

### Identification of Differentially Expressed Genes

The expression of genes was calculated using the FPKM (fragments per kb per million reads) method (Li & Dewey, 2011). |log2(FoldChange)| > 1.5 & *p*-value < 0.05 were set to be the hypergeometric distribution threshold for significantly different expression levels. All DEGs were conducted the GO functional and KEGG pathway enrichment analysis using the GO and KEGG databases.

### Validation by Quantitative Real-Time PCR

Total RNA was extracted from gonads, and cDNA was obtained by reverse transcription conducted by PrimeScriptTM RT reagent Kit with gDNA Eraser (Takara) following the manufacturer’s protocol. We investigated the significant expression difference between two groups of genes selected based on related pathways by qPCR. Three candidate internal reference genes—beta actin, 18 S, and gapdh—were screened out to test for the stability of expression. The cDNA templates of two group’s samples were taken in equal quantities and diluted by five gradients successively after mixing; the concentrations were 1, 1/10, 1/100, 1/1 000, 1/10,000, and 1/100,000 times. qPCR was performed on the products, and each reaction was carried out in triplicate. The standard curve was drawn with the CT value as the ordinate and the logarithm of dilution multiple as the abscissa using Microsoft Excel 2010. We selected gapdh as the reference gene; it shows a good linear relationship between template concentration and CT value (R^2^ = 0.996). The relative expression levels of differential genes were calculated by the 2^-∆∆CT^ method. Primers were designed using Primer Premier five software (Premier Biosoft, United States) on the basis of sequences obtained from the transcriptome result with the primers listed in Supplementary Table S1. The amplification program was performed as 95°Cfor 30 s followed by 95°Cfor 5 s and 60°C for 30 s (40 cycles).

### Statistical Analysis

The data were presented as mean ± standard error (SE). Significant differences were established for all results using T-tests. The value of a statistical significance was regarded as *p* < 0.05. All statistical analyses were performed using the SPSS software package (version 26.0).

## Results

### Isolation of Crab Females With Stage IV Ovaries by the Device

**FIGURE 2:**
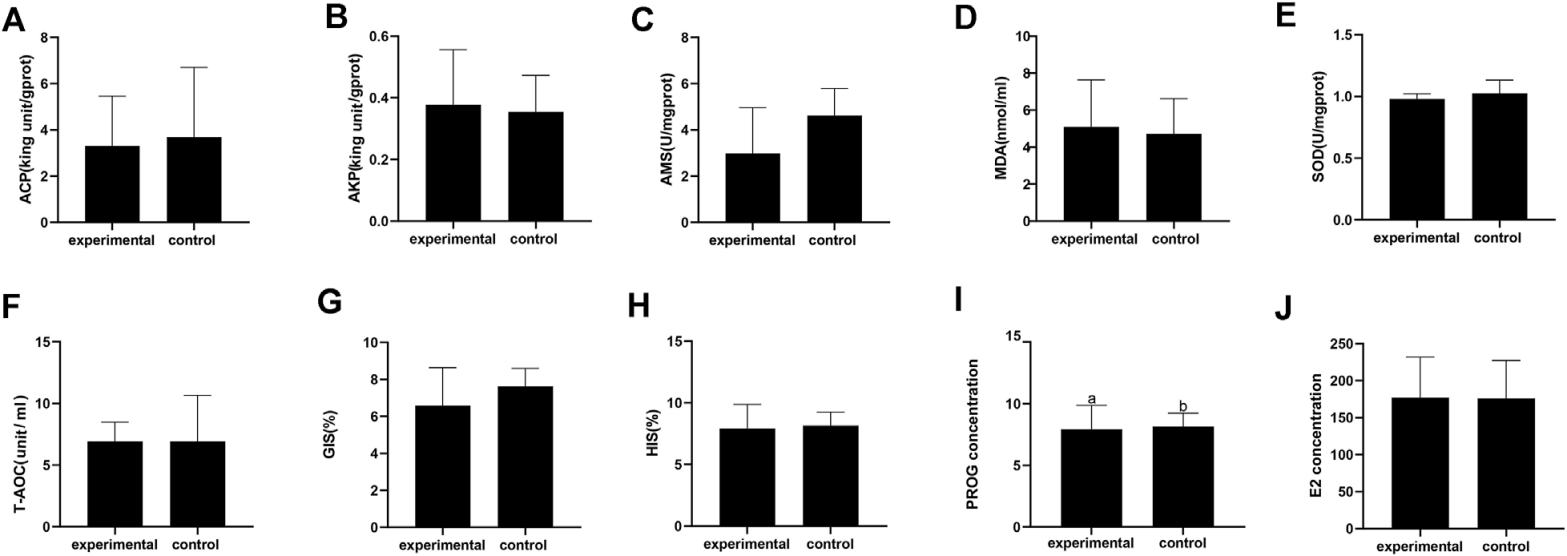
Physiological and biochemical index of Eriocheir sinensis in experimental and control group. **(A)** Acid phosphatase; **(B)** Alkaline phosphatase; **(C)** Serum amylase; **(D)** Malondialdehyde; **(E)** Superoxide dismutase; **(F)** Total antioxidant capacity; **(G)** Gonadosomatic index; **(H)** Hepatosomatic index; **(I)** Progesterone content in serum; **(J)** Estradiol content in serum.

**FIGURE 3:**
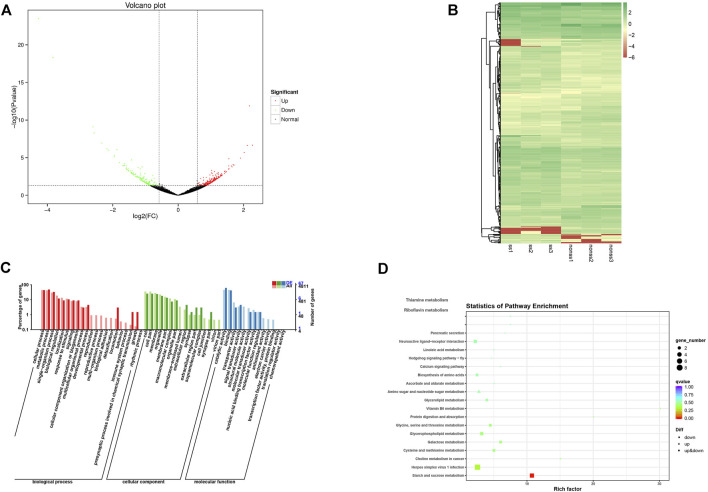
Isolation result chart. **(A)** Test crabs screened out with device; **(B)** Test crabs not screened out with device; **(C)** Ovarian section in stage IV of test crabs screened out; **(D)** Ovarian section in stage III of test crabs not screened out.

Some crabs were isolated by the device while some were not (Figure 2A,B). In this experiment, 10 crabs were isolated out of 40. Based on the previous study, ovarian stages were divided into five stages. In ovarian biopsies of crabs in the experimental group, the oocytes were squeezed into a honeycomb and accumulated a larger yolk (Figure 2C). In contrast, the oocytes were oval and the deposition size of yolk granules was smaller in the control group (Figure 2D). Ovaries of subjects in the experimental group were at stage IV, while those of the control groups were at stage III. The concentration of PROG in the experimental group was significantly reduced compared to the control group (*p <* 0.05), but no significant difference was detected in E2 (Figure 3I,J).

### Physiological and Biochemical Parameters and Fatty Acid Profiles in Response to Identified Ovaries of Different Developmental Stages Using This Device

There was no significant difference in AMS, T-AOC, SOD, ACP, AKP, and MDA between the isolated group and the control group (Figure 3A–F). Moreover, no significant differences in the HIS and GIS were detected between the two groups (Figure 3G,H).

Fatty acid compositions in the gonads of two groups are presented in Table 1. A total of 26 fatty acids were detected. In terms of individual fatty acids, only the stearic acid (C18:4) was significantly different between the groups (*p* < 0.05). In addition, no significant difference was detected in saturated fatty acids (SAF), monounsaturated fatty acids (MUFA), polyunsaturated fatty acids (PUFA), and highly unsaturated fatty acids (HUFA).

**TABLE 1:** Fatty acid composition in gonad of two groups.

Fatty acid	Experimental group	Control group
C10:0	0.018 ± 0.001	0.018 ± 0.001
C12:0	0.170 ± 0.159	0.089 ± 0.062
C14:0	1.388 ± 0.244	1.166 ± 0.076
C15:0	0.369 ± 0.081	0.318 ± 0.027
C16:0	14.247 ± 0.445	14.453 ± 0.195
C17:0	0.311 ± 0.055	0.239 ± 0.032
C18:0	3.445 ± 0.358	3.104 ± 0.514
C20:0	0.113 ± 0.030	0.094 ± 0.011
C22:0	0.053 ± 0.023	0.055 ± 0.003
∑SFA	20.113 ± 0.694	19.536 ± 0.477
C14:1	0.166 ± 0.049	0.146 ± 0.020
C16:1	14.247 ± 0.445	12.062 ± 1.545
C17:1	0.819 ± 0.166	0.754 ± 0.068
C18:1	28.815 ± 2.259	30.188 ± 1.492
C20:1	1.780 ± 0.605	1.658 ± 0.466
C22:1	0.594 ± 0.207	0.536 ± 0.207
∑MUFA	42.996 ± 1.028	45.344 ± 0.937
C18:2	8.100 ± 1.213	7.423 ± 2.063
C20:2	0.650 ± 0.065	0.675 ± 0.096
C18:3	1.531 ± 0.0926	1.706 ± 0.203
C20:3	0.194 ± 0.039	0.160 ± 0.020
C22:3	0.523 ± 0.051	0.527 ± 0.063
C18:4	0.703 ± 0.005^a^	0.591 ± 0.030^b^
C20:4	2.738 ± 0.201	2.461 ± 0.379
C22:4	0.213 ± 0.024	0.2 ± 0.031
C20:5	9.108 ± 1.052	8.236 ± 0.679
C22:5	0.687 ± 0.127	0.63 ± 0.154
C22:6	12.445 ± 1.889	12.510 ± 1.487
∑PUFA	36.891 ± 1.712	35.119 ± 0.494
∑HUFA	25.908 ± 2.528	24.724 ± 1.609

Value are present as means ± SE, Means with different superscript in the same row are significantly different (*p* < 0.05). ∑SFA, total saturated fatty acid; ∑MUFA, total monounsaturated fatty acid; ∑PUFA, total polyunsaturated fatty acid; ∑HUFA, total highly unsaturated fatty acid.

### Transcriptional Levels in Answer to Isolation

**FIGURE 4:**
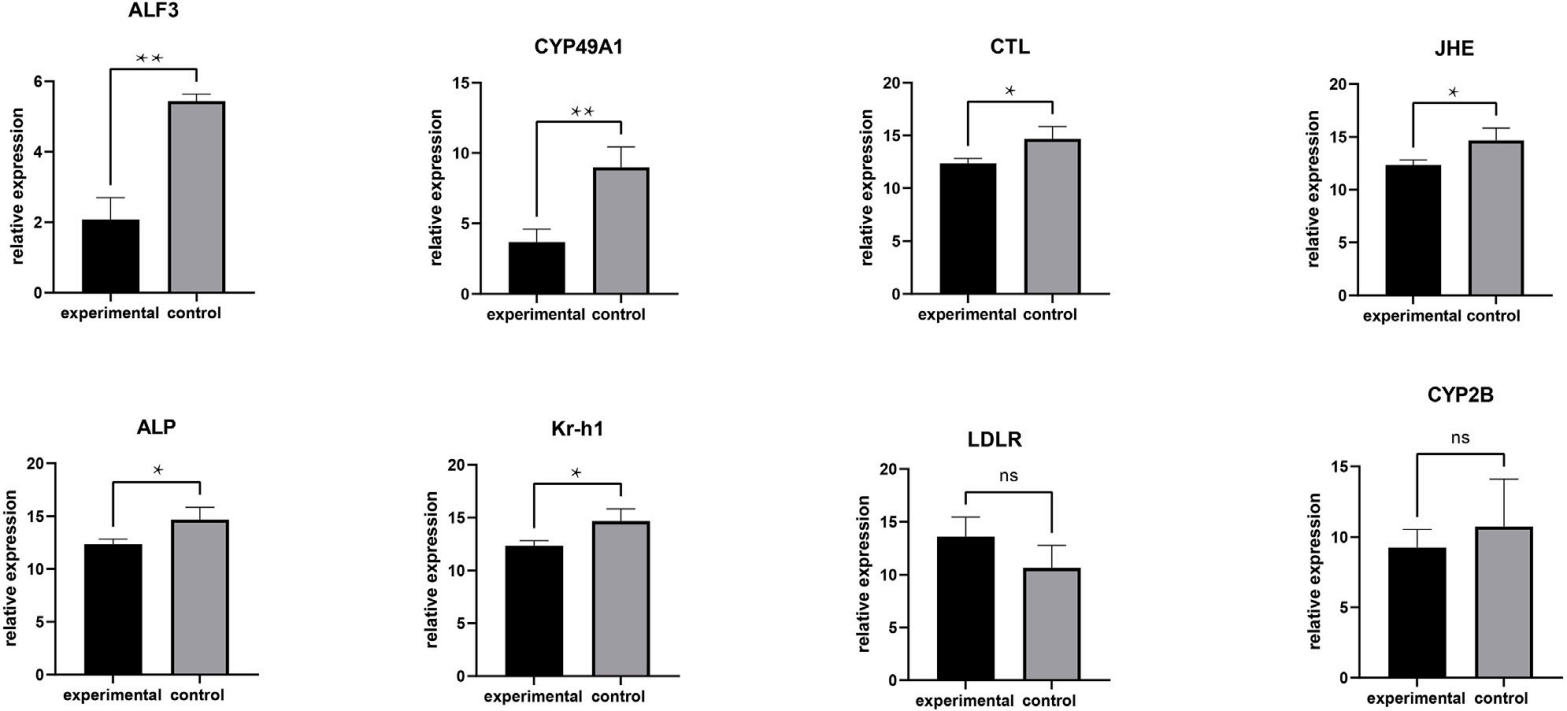
RT-PCR analysis of DEGs. **means greatly significant differences (*p* < 0.01), *means significant differences (*p* < 0.05) and ns means no significant difference.

In total 40.18 GB of clean data were obtained after the sequencing quality control, and the percentage Q30 base of each sample was not less than 91.83%. Clean data were conducted through sequence alignment using the specified genome as a reference (https://www.ncbi.nlm.nih.gov/genome/?term=Eriocheir+sinensis), with the comparative efficiency ranging from 63.60 to 68.83% (Supplementary Table.S2). A total of 15,246 new genes were discovered by splicing mapped reads. The volcano plots present the distribution of the differentially expressed genes, according to the FDR≤ 0.001 and Log Fold change threshold (Figure 4A). A total of 388 differentially expressed genes (DEGs) were identified by differential expression analysis between two groups with 233 upregulated genes and 155 downregulated genes. A heatmap was plotted to illustrate the relative expression profiles of the DEGs in the two groups (Figure 4B). The trend of gene expression was different between the two groups, while the trend of gene expression was consistent between the groups.

Functional annotation and enrichment analysis of differentially expressed genes were carried out. The Gene Ontology (GO) annotation system contains three main branches: biological process, molecular function, and cellular component. Within the biological process category, the proportion of functional classification of differentially expressed genes (DEGs) and all genes related to locomotion, the presynaptic process involved in chemical synaptic transmission, and growth showed significant differences. Moreover, the annotation genes were partially associated with reproduction, the reproductive process, and the multi-organism process but none of DEGs. In the cellular component category and molecular function category, the proportion of genes associated with the membrane-enclosed lumen and structural molecule activity showed notable differences between DEGs and all annotation genes (Figure 4C).

Enrichment analysis of the KEGG pathways of the differentially expressed genes showed the top 20 pathways with the lowest significant Q values. The number of differentially expressed genes enriched in herpes simplex virus one infection pathways was the largest followed by the starch and sucrose metabolism pathways. Furthermore, the glycerophospholipid metabolism pathway and neuroactive ligand-receptor interaction pathway enriched many differentially expressed genes (Figure 4D).

**FIGURE 5:**
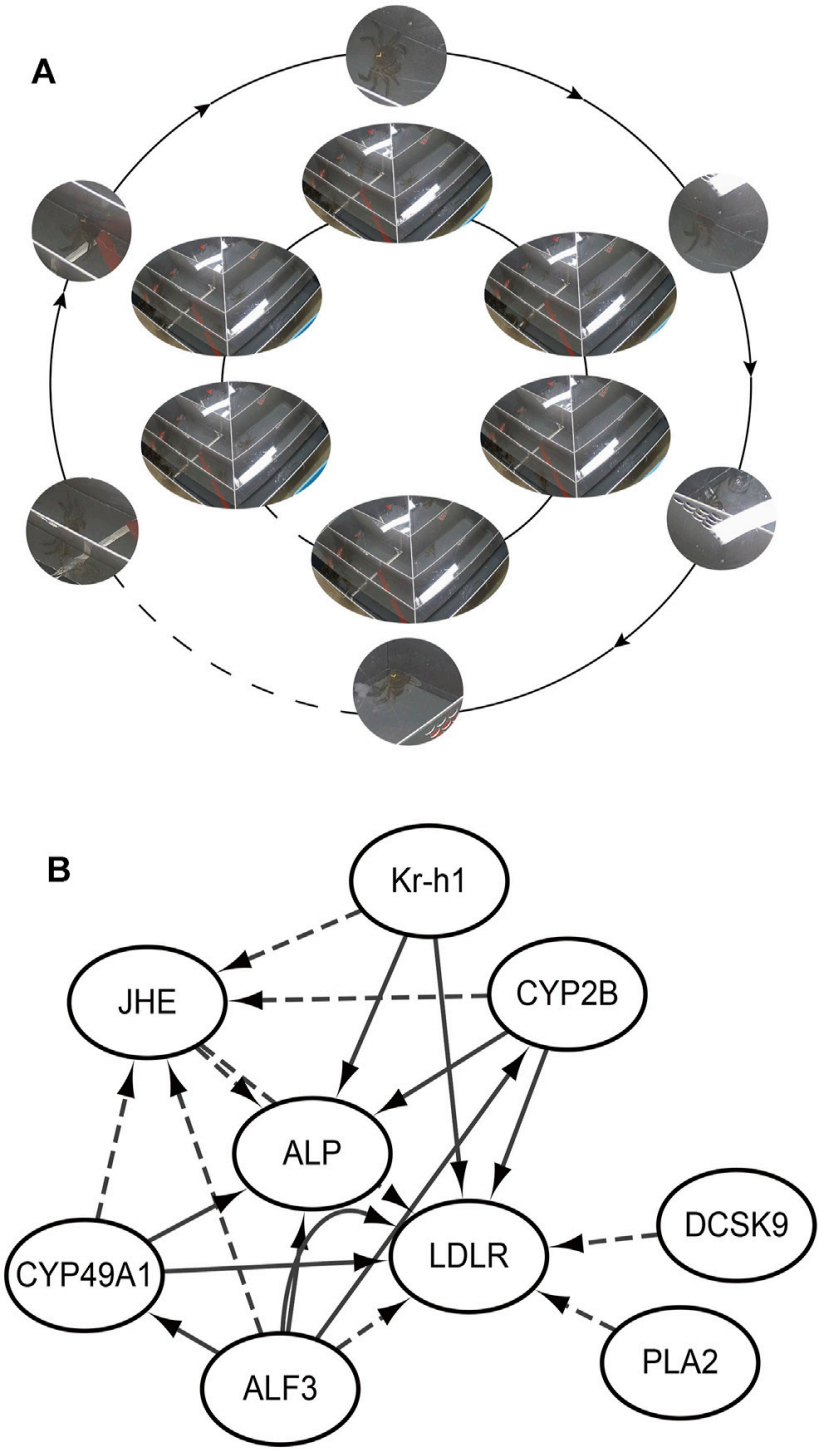
Isolation behavior from internal mechanisms. **(A)** Realizing isolation by the female passing through the screening plate and moving closer to the male; **(B)** The internal mechanism by which female crabs approach male crabs; ALP: alkaline phosphatase; LDLR: low-density lipoprotein receptor; Kr-h1: krueppel homolog 1; CYP2B: cytochrome P450 CYP2B; JHE: juvenile hormone esterase; CTL: c-type lectin; CYP49A1: cytochrome P450 49a1; ALF3: anti-lipopolysaccharide factor 3; PLA2: Phospholipase A2. The solid line is promotion and the dashed line is inhibition.

**FIGURE 6:**
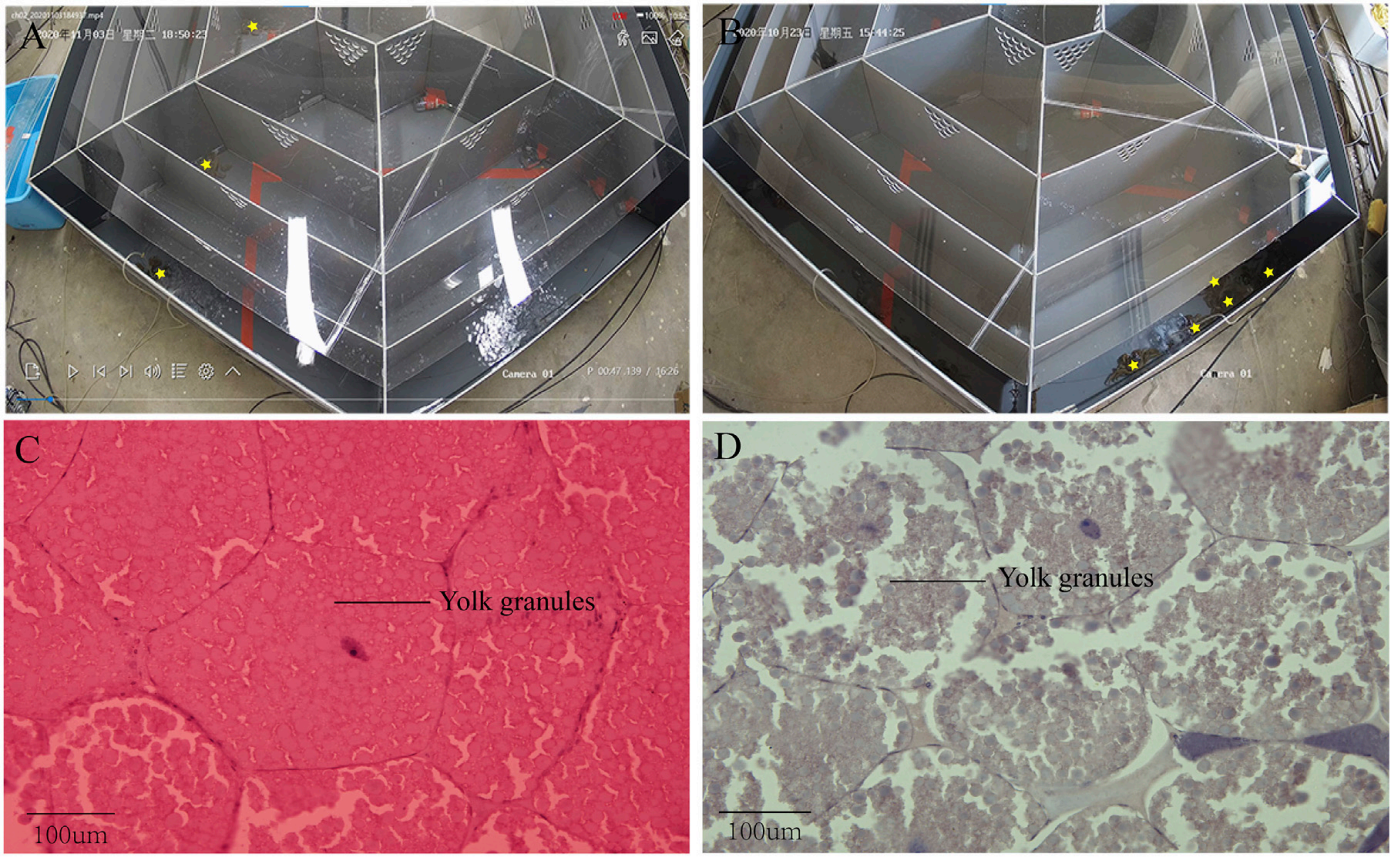
**(A)** Volcano plot of DEGs; **(B)** Heat map summarizing DEGs identified from different sample; **(C)** GO functional classification of DEGs; **(D)** KEGG of annotated of DEGs.

To validate the RNA-seq findings, we chose eight genes that were significantly different in the DEG analysis and performed RT-PCR: alkaline phosphatase (ALP), low-density lipoprotein receptor (LDLR), krueppel homolog 1 (Kr-h1), cytochrome P450 CYP2B (CYP2B), juvenile hormone esterase (JHE), c-type lectin (CTL), cytochrome P450 49a1 (CYP49A1), and anti-lipopolysaccharide factor 3 (ALF3). The RT-PCR results were similar to those of RNA-seq (Figure 5). Meanwhile, these DEGs are associated with gonadal development and related to each other, among which LDLR functions a key role in oocyte development showing the internal mechanism of isolation between different gonadal development in the two groups of crabs (Figure 6).

## Discussion

### Estimate of Gonadal Development Status by Sexual Motivation

Pheromones are special social chemical signals used for within-species communication, and they are used to reliably indicate the reproductive status of members of other social groups (Spencer et al., 2004), which prime for sexual motivation regulated by the nervous system (Schecklmann et al., 2015). Human pheromones are mainly derived from apocrine glands in the armpit and pudendal regions, which do not come into play until they are sexually mature (Grammer et al., 2005). Therefore, it is feasible to judge the gonad development state by sexual motivation.

Gonadal hormones play a key role in the sexual differentiation of the brain and behavior (Jennings & de Lecea, 2020). Men and women who are in love experience gonadal hormone changes (Seshadri, 2016; Sorokowski et al., 2019). The sequential rise in estrogen and progesterone promotes the production of sexual motivation through the brain and physiology (Wallen, 2001). Previous investigation has also indicated that estrogens such as estradiol (E2) and progesterone (PROG) are closely related to ovarian development (Merlin et al., 2015; Liu et al., 2018). The PROG concentrations in the hemolymph increase significantly in the early stage of ovarian development, reaching a maximum at stage III, and then decrease gradually in Chinese mitten crabs (Wu et al., 2014). In this investigation, the concentration of PROG in experimental groups was significantly reduced compared with the control group. The concentration of PROG shows a downward trend between stage III and stage IV, which is in line with the previous investigation.

Similarly, the concentration of E2 increases significantly in the early stage of ovarian development and drops later on (Pan et al., 2018). Nevertheless, no significant difference was detected in E2 in this investigation. The current findings suggest that there are transient hormonal changes when people fall in love (Marazziti & Canale, 2004). Scholars have found that the E2 concentration fluctuates during the estrus cycle in both vertebrates and invertebrates (Rota et al., 2007; Sharma et al., 2014; Singh et al., 2016). In this investigation, female crabs with mature gonads were isolated within half an hour through estrus by which female crabs are attracted to male crabs. Therefore, probably due to the presence of male crabs, estrus in female crabs causes an increase in E2. From another point of view, estradiol may act as a sex pheromone to induce a preference for the male pheromone in the Chinese mitten crab.

### Temporal and Spatial Principles for the Device

The opposite sexes judge opposite sexual receptivity through sexual pheromone; they depend on the hypothalamus to further produce sexual motivation and complete reproductive activities. Some scholars have found that the hypothalamic-related receptors in rats changed with the time of pheromone exposure (Grigorjev & Munaro, 1999). Moreover, the gonadotropin-releasing hormone pulse generator has a refractory period to pheromone signals, which indicates exposed pheromones have certain timeliness (Murata et al., 2011). Therefore, in the present study, each isolation test is controlled within half an hour to ensure accuracy of the results.

The Fibonacci sequence has been found in many fields, including art, nature, and architecture. Diffusion, ubiquitous in life, proceeds faster than what is ordinary in the Fibonacci sequence (Boettcher & Gonçalves, 2008). In the present study, several channels whose widths increase following the Fibonacci sequence were set up with screening plates from the outside to the inside of the device, which probably promotes pheromone diffusion to accelerate the recognition of the opposite sex. Meanwhile, the increasing distance increases the difficulty of crossing to separate physically and physiologically improved individuals to some extent.

### The Role of Fatty Acids in Gonadal Development

During ovarian development, nutrients are transported through the blood to the developing ovaries (He et al., 2014). The dietary regime has a great impact on ovarian development and reproductive performance, especially so for dietary lipid (Ribeiro et al., 2012), among which the ratio between n-3PUFA and n-6PUFA immensely impacts ovarian maturation. An appropriate proportion can promote ovarian development and improve fecundity to a certain extent. Sexual maturation is accompanied by the release of pheromones, which promotes the sex motivation of the opposite sex.

In the component analysis of fatty acids, the levels of stearic acid (C18:4) in the isolated group were significantly higher than that of the eliminated group, which indicates that ovaries probably had a high requirement for stearic acid at a later stage of ovarian development. It is known that stearic acid makes a difference to prostaglandin biosynthesis and metabolism (Ogborn et al., 2003). Meanwhile, studies have shown that prostaglandins are essential to reproductive maturation and ovulation (Fujimori et al., 2011). For instance, the number and the diameter of the oocytes were significantly increased by the addition of PGE_2_ and PGF_2а_ into ovaries (Reddy et al., 2004). Furthermore, the level of PGE_2_ and PGF_2а_ in the ovary and hemolymph of female shrimp fluctuated with the stage of ovarian development in penaeid shrimp (Prasertlux et al., 2011; Wimuttisuk et al., 2013). Therefore, significantly higher levels of stearic acid in the isolated group may be the ovulation of mature gonads. At present, there is no relevant research on stearic acid, whose function needs to be further explored.

### Key Genes Related to Gonadal Development

The isolation of Chinese mitten crabs with mature gonads was based on the principle of pheromone to produce behavioral changes, whose biosynthesis depends on the regulation of neuropeptides (Torfs et al., 2001) demonstrated in pathway enrichment analysis. Furthermore, GO and KEGG enrichment analysis showed that numerous DEGs have a significant correlation with gonadal development. Anti-lipopolysaccharide factors have been proved to be able to induce the expression of various cytochrome P450s (Ashida et al., 2003). The synthesis of estrogens and oocyte development is dependent on the activity of cytochrome enzyme P450 aromatase, which is a terminal enzyme in the estrogen biosynthetic pathway (Conley & Hinshelwood, 2001; Wen et al., 2014). Previous investigations have shown that some 450 s participate in the biosynthesis and metabolism of the juvenile hormone (Zhou et al., 2006). In addition, the juvenile hormone can induce vitellogenin receptor (VgR) expression (Liu et al., 2019), which is a member of the low-density lipoprotein receptor (Lu et al., 2015). Vitellogenin (Vg) as the precursor of the major yolk protein vitellin (Vn) has a key role in oocyte development (Lee et al., 2008). Vg in hemolymphs was sequestered into the developing oocytes *via* endocytosis that vitellogenin receptor (VgR) mediates. Thus it can be seen that the developmental dyssynchrony and the effectiveness of the isolation were demonstrated from the perspective of genes isolated from crabs and unisolated crabs.

In the case of strong market demand, the fastest and most effective way to judge the gonadal development state is by the external characteristics and behavior of the individual. Studies have shown no correlation between external morphology and gonadal development state. However, members of the opposite sex with mature gonads secrete pheromones to encourage sexual motivation to facilitate proximity through nerve conduction, which can realize individual separation with varying degrees of gonadal development. The design of the separation device follows the time rule of the pheromone effect and the spatial principle of rapid propagation, which takes into account the rapid and effective transmission of pheromones. The relationship between sex hormones and gonadal development was further confirmed by the determination of sex hormones. DEGs showed potential differences in gonadal development between the two groups in terms of internal mechanisms. The effect of fatty acids on gonadal development was further explored by detecting the difference in gonad fatty acid composition, which provided a reference value for breeding Chinese mitten crabs of higher quality.

Overall, we used the device to realize the rapid isolation of Chinese mitten crabs with mature gonads. Whether from internal genes or external ovarian morphology and composition, ovarian development in the experimental group was higher than that in the control group and was nearly fully mature. Not only can this method facilitate the breeding of Chinese mitten crabs, but it also provides a model for the rapid determination of their commercial value. Environmental factors can regulate pheromone secretion (Wang et al., 2021), and the environmental conditions of the screening process need to be further investigated to speed up the isolation process.

## Data Availability

The original contributions presented in the study are publicly available. This data can be found here: ncbi.nlm.nih.gov/bioproject, PRJNA805383.

## References

[B1] Ashburner M. Ball C. A. Blake J. A. Botstein D. Butler H. Cherry J. M. (2000). Gene Ontology: Tool for the Unification of Biology. Nat. Genet. 25, 25–29. 10.1038/75556 10802651 PMC3037419

[B2] Ashida H. Hashimoto T. Nonaka Y. Fukuda I. Kanazawa K. Danno G.-i. (2003) Suppression of Cytochrome P4501 a Subfamily in Mouse Liver by Oral Intake of Polysaccharides from Mushrooms, Lentinus Edodes and Agaricus Blazei. In Food Factors in Health Promotion and Disease Prevention, Shahidiu F. Ho C. T. Watanabe S. Osawa T. (eds.) pp 235–248. 10.1021/bk-2003-0851.ch021

[B3] Barry K. L. (2010). Influence of Female Nutritional Status on Mating Dynamics in a Sexually Cannibalistic Praying Mantid. Anim. Behav. 80, 405–411. 10.1016/j.anbehav.2010.05.024

[B4] Boettcher S. Gonçalves B. (2008). Anomalous Diffusion on the Hanoi Networks. EPL (Europhysics Letters) 84 (3). 10.1209/0295-5075/84/30002

[B5] Conley A. Hinshelwood M. (2001). Mammalian Aromatases. Reproduction 121, 685–695. 10.1530/rep.0.1210685 11427156

[B6] Fujimori C. Ogiwara K. Hagiwara A. Rajapakse S. Kimura A. Takahashi T. (2011). Expression of Cyclooxygenase-2 and Prostaglandin Receptor EP4b mRNA in the Ovary of the Medaka Fish, *Oryzias latipes*: Possible Involvement in Ovulation. Mol. Cell Endocrinol. 332, 67–77. 10.1016/j.mce.2010.09.015 20932877

[B7] Grammer K. Fink B. Neave N. (2005). Human Pheromones and Sexual Attraction. Eur. J. Obstet. Gynecol. Reprod. Biol. 118, 135–142. 10.1016/j.ejogrb.2004.08.010 15653193

[B8] Grigorjev C. Munaro N. (1999). Time-dependent GABA-Ergic Activity in Olfactory Bulb and Hypothalamus of Proestrous Rats. Brain Res. Bull. 48, 569–572. 10.1016/s0361-9230(98)00148-8 10386836

[B9] Harrison M. A. Shortall J. C. (2011). Women and Men in Love: Who Really Feels it and Says it First? J. Soc. Psychol. 151, 727–736. 10.1080/00224545.2010.522626 22208110

[B10] He J. Wu X. Li J. Huang Q. Huang Z. Cheng Y. (2014). Comparison of the Culture Performance and Profitability of Wild-Caught and Captive Pond-Reared Chinese Mitten Crab (Eriocheir Sinensis) Juveniles Reared in Grow-Out Ponds: Implications for Seed Selection and Genetic Selection Programs. Aquaculture 434, 48–56. 10.1016/j.aquaculture.2014.07.022

[B11] Herborg L.-M. Bentley M. G. Clare A. S. Last K. S. (2006). Mating Behaviour and Chemical Communication in the Invasive Chinese Mitten Crab Eriocheir Sinensis. J. Exp. Mar. Biol. Ecol. 329, 1–10. 10.1016/j.jembe.2005.08.001

[B12] Jennings K. J. de Lecea L. (2020). Neural and Hormonal Control of Sexual Behavior. Endocrinology 161 (10). 10.1210/endocr/bqaa150 PMC7507403 32845294

[B13] Kamio M. Schmidt M. Germann M. W. Kubanek J. Derby C. D. (2014). The Smell of Moulting: N-Acetylglucosamino-1,5-Lactone Is a Premoult Biomarker and Candidate Component of the Courtship Pheromone in the Urine of the Blue Crab, *Callinectes sapidus* . J. Exp. Biol. 217, 1286–1296. 10.1242/jeb.099051 24363413

[B14] Kamio M. Araki M. Nagayama T. Matsunaga S. Fusetani N. (2005). Behavioral and Electrophysiological Experiments Suggest that the Antennular Outer Flagellum Is the Site of Pheromone Reception in the Male Helmet Crab Telmessus Cheiragonus. Biol. Bull. 208, 12–19. 10.2307/3593096 15713808

[B15] Lee K.-W. Hwang D.-S. Rhee J.-S. Ki J.-S. Park H. G. Ryu J.-C. (2008). Molecular Cloning, Phylogenetic Analysis and Developmental Expression of a Vitellogenin (Vg) Gene from the Intertidal Copepod Tigriopus Japonicus. Comp. Biochem. Physiol. B: Biochem. Mol. Biol. 150, 395–402. 10.1016/j.cbpb.2008.04.009 18539492

[B16] Li B. Dewey C. N. (2011). RSEM: Accurate Transcript Quantification from RNA-Seq Data with or without a Reference Genome. Bmc Bioinformatics 12, 323. 10.1186/1471-2105-12-323 21816040 PMC3163565

[B17] Liu M. Pan J. Liu Z. Cheng Y. Gong J. Wu X. (2018). Effect of Estradiol on Vitellogenesis and Oocyte Development of Female Swimming Crab, Portunus Trituberculatus. Aquaculture 486, 240–245. 10.1016/j.aquaculture.2017.12.034

[B18] Liu W. Guo S. Sun D. Zhu L. Zhu F. Lei C.-L. (2019). Molecular Characterization and Juvenile Hormone-Regulated Transcription of the Vitellogenin Receptor in the Cabbage Beetle Colaphellus Bowringi. Comp. Biochem. Physiol. A: Mol. Integr. Physiol. 229, 69–75. 10.1016/j.cbpa.2018.12.004 30553881

[B19] Long C. P. Antoniewicz M. R. (2014). Quantifying Biomass Composition by Gas Chromatography/Mass Spectrometry. Anal. Chem. 86, 9423–9427. 10.1021/ac502734e 25208224 PMC5842031

[B20] Lu H. Yuan G. Zhang J. Liu G. (2020). Recognition of Impulse of Love at First Sight Based on Photoplethysmography Signal. Sensors (Basel) 20 (22). 10.3390/s20226572 PMC7698503 33213065

[B21] Lu K. Shu Y. Zhou J. Zhang X. Zhang X. Chen M. (2015). Molecular Characterization and RNA Interference Analysis of Vitellogenin Receptor from Nilaparvata Lugens (Stål). J. Insect Physiol. 73, 20–29. 10.1016/j.jinsphys.2015.01.007 25617689

[B22] Marazziti D. Canale D. (2004). Hormonal Changes when Falling in Love. Psychoneuroendocrinology 29, 931–936. 10.1016/j.psyneuen.2003.08.006 15177709

[B23] McCarthy E. A. Naik A. S. Coyne A. F. Cherry J. A. Baum M. J. (2018). Effect of Ovarian Hormones and Mating Experience on the Preference of Female Mice to Investigate Male Urinary Pheromones. Chem. Senses 43, 97–104. 10.1093/chemse/bjx073 29211837 PMC5863565

[B24] Merlin J. Mohanlal D. L. Balasubramanian C. P. Sherly T. Subramoniam T. Syamadayal J. (2015). Induction of Vitellogenesis and Reproductive Maturation in Tiger Shrimp,Penaeus Monodonby 17ß-Estradiol and 17α-Hydroxyprogesterone:in Vivoandin Vitrostudies. Invertebrate Reprod. Develop. 59, 166–175. 10.1080/07924259.2015.1051192

[B25] Murata K. Wakabayashi Y. Sakamoto K. Tanaka T. Takeuchi Y. Mori Y. (2011). Effects of Brief Exposure of Male Pheromone on Multiple-Unit Activity at Close Proximity to Kisspeptin Neurons in the Goat Arcuate Nucleus. J. Reprod. Develop. 57, 197–202. 10.1262/jrd.10-070e 21123964

[B26] Ogborn M. R. Nitschmann E. Bankovic-Calic N. Weiler H. A. Fitzpatrick-Wong S. Aukema H. M. (2003). Dietary Conjugated Linoleic Acid Reduces PGE2 Release and Interstitial Injury in Rat Polycystic Kidney Disease. Kidney Int. 64, 1214–1221. 10.1046/j.1523-1755.2003.00215.x 12969139

[B27] Pan J. Liu M. Chen T. Cheng Y. Wu X. (2018). Immunolocalization and Changes of 17beta-Estradiol during Ovarian Development of Chinese Mitten Crab Eriocheir Sinensis. Cell Tissue Res 373, 509–520. 10.1007/s00441-018-2834-x 29707750

[B28] Prasertlux S. Sittikankaew K. Chumtong P. Khamnamtong B. Klinbunga S. (2011). Molecular Characterization and Expression of the Prostaglandin Reductase 1 Gene and Protein during Ovarian Development of the Giant Tiger Shrimp *Penaeus monodon* . Aquaculture 322-323, 134–141. 10.1016/j.aquaculture.2011.09.037

[B29] Ribeiro K. Franceschini-Vicentini I. B. Papa L. P. New M. B. Valenti W. C. (2012). Effect of Polyunsaturated Fatty Acids on the Fecundity of the Amazon River prawnMacrobrachium amazonicum(Heller, 1862). Aquac. Res. 43, 1756–1763. 10.1111/j.1365-2109.2011.02980.x

[B30] Rota A. Veronesi M. C. Volpe S. Riccardi A. Battocchio M. (2007). Estradiol-17beta, Progesterone and Testosterone Plasma Concentrations during Estrus in the Bitch. Vet. Res. Commun. 31 Suppl 1, 197–199. 10.1007/s11259-007-0031-6 17682874

[B31] Schecklmann M. Engelhardt K. Konzok J. Rupprecht R. Greenlee M. W. Mokros A. (2015). Sexual Motivation Is Reflected by Stimulus-dependent Motor Cortex Excitability. Soc. Cogn. Affect Neurosci. 10, 1061–1065. 10.1093/scan/nsu157 25556214 PMC4526478

[B32] Seshadri K. (2016). The Neuroendocrinology of Love. Indian J. Endocr. Metab. 20, 558–563. 10.4103/2230-8210.183479 PMC4911849 27366726

[B33] Shao L. Wang C. He J. Wu X. Cheng Y. (2013). Hepatopancreas and Gonad Quality of Chinese Mitten Crabs Fattened with Natural and Formulated Diets. J. Food Qual. 36, 217–227. 10.1111/jfq.12030

[B34] Shao L. Wang C. He J. Wu X. Cheng Y. (2014). Meat Quality of Chinese Mitten Crabs Fattened with Natural and Formulated Diets. J. Aquat. Food Product. Tech. 23, 59–72. 10.1080/10498850.2012.694583

[B35] Sharma S. Nigam R. Pandey V. Ghuman S. S. Singh P. (2014). Circulating levels of estradiol 17-β and progesterone vis à vis nitric oxide and nitric oxide synthase at and around oestrus in cycling buffaloes. J. Appl. Anim. Res. 43, 214–217. 10.1080/09712119.2014.963090

[B36] Shimomura K. Akasaka K. Yajima A. Mimura T. Yajima S. Ohsawa K. (2010). Contact Sex Pheromone Components of the Seed Beetle, Callosobruchus Analis (F.). J. Chem. Ecol. 36, 955–965. 10.1007/s10886-010-9841-z 20697783

[B37] Singh M. D. Morris M. J. Guimarães D. A. Bourne G. Garcia G. W. (2016). Serological Evaluation of Ovarian Steroids of Red-Rumped agouti (*Dasyprocta leporina*) during the Estrous Cycle Phases. Anim. Reprod. Sci. 175, 27–32. 10.1016/j.anireprosci.2016.10.005 27876215

[B38] Sorokowski P. Żelaźniewicz A. Nowak J. Groyecka A. Kaleta M. Lech W. (2019). Romantic Love and Reproductive Hormones in Women. Int. J. Environ. Res. Public Health 16 (21). 10.3390/ijerph16214224 PMC6861983 31683520

[B39] Spencer N. A. McClintock M. K. Sellergren S. A. Bullivant S. Jacob S. Mennella J. A. (2004). Social Chemosignals from Breastfeeding Women Increase Sexual Motivation. Horm. Behav. 46, 362–370. 10.1016/j.yhbeh.2004.06.002 15325237

[B40] Sreenivasula Reddy P. Ramachandra Reddy P. Purna Chandra Nagaraju G. (2004). The Synthesis and Effects of Prostaglandins on the Ovary of the Crab Oziotelphusa Senex Senex. Gen. Comp. Endocrinol. 135, 35–41. 10.1016/j.ygcen.2003.08.002 14644642

[B41] Thiel M. Breithaupt T. (2011). Chemical Communication in Crustaceans Research Challenges for the Twenty-First Century 5152. 10.1007/978-0-387-77101-4(2011)3-22

[B42] Torfs P. Nieto J. Cerstiaens A. Boon D. Baggerman G. Poulos C. (2001). Pyrokinin Neuropeptides in a Crustacean. Eur. J. Biochem. 268, 149–154. 10.1046/j.1432-1327.2001.01858.x 11121115

[B43] Varner E. Gries R. Takács S. Fan S. Gries G. (2018). Identification and Field Testing of Volatile Components in the Sex Attractant Pheromone Blend of Female House Mice. J. Chem. Ecol. 45, 18–27. 10.1007/s10886-018-1032-3 30411204

[B44] Wallen K. (2001). Sex and Context: Hormones and Primate Sexual Motivation. Horm. Behav. 40, 339–357. 10.1006/hbeh.2001.1696 11534996

[B45] Wang G. Vega-Rodríguez J. Diabate A. Liu J. Cui C. Nignan C. (2021). Clock Genes and Environmental Cues Coordinate Anopheles Pheromone Synthesis, Swarming, and Mating. Science 371, 411–415. 10.1126/science.abd4359 33479155 PMC9854397

[B46] Wang S. He Y. Wang Y. Tao N. Wu X. Wang X. (2016). Comparison of Flavour Qualities of Three Sourced Eriocheir Sinensis. Food Chem. 200, 24–31. 10.1016/j.foodchem.2015.12.093 26830556

[B47] Wen H.-S. Mu W.-J. Yang Y.-P. Shi D. He F. Li J.-F. (2014). Molecular Physiology Mechanism of Cytochrome P450 Aromatase-Regulating Gonad Development in Ovoviviparous Black Rockfish (Sebastes Schlegeli). Aquac. Res. 45, 1685–1696. 10.1111/are.12114

[B48] Wierman M. E. (2007). Sex Steroid Effects at Target Tissues: Mechanisms of Action. Adv. Physiol. Edu. 31, 26–33. 10.1152/advan.00086.2006 17327579

[B49] Wimuttisuk W. Tobwor P. Deenarn P. Danwisetkanjana K. Pinkaew D. Kirtikara K. (2013). Insights into the Prostanoid Pathway in the Ovary Development of the Penaeid Shrimp *Penaeus monodon* . PLoS One 8, e76934. 10.1371/journal.pone.0076934 24116186 PMC3792876

[B50] Wu X. Chen H. Liu Z. Cheng Y. (2014). Immunorecognition and Distribution of Progesterone Receptors in the Chinese Mitten CrabEriocheir sinensisDuring Ovarian Development. J. Shellfish Res. 33, 35–43. 10.2983/035.033.0105

[B51] Wyatt T. D. (2010). Pheromones and Signature Mixtures: Defining Species-Wide Signals and Variable Cues for Identity in Both Invertebrates and Vertebrates. J. Comp. Physiol. A: Neuroethol. Sens. Neural Behav. Physiol. 196 (10), 685–700. 20680632 10.1007/s00359-010-0564-y

[B52] Wyatt T. D. (2015). The Search for Human Pheromones: The Lost Decades and the Necessity of Returning to First Principles. Proceedings of the Royal Society B-Biological Sciences. 282 (1804). 10.1098/rspb.2014.2994 PMC4375873 25740891

[B53] Yambem H. Shindo M. Yamazaki F. (1999). A Releaser Pheromone that Attracts Males in the Urine of Mature Female Masu salmon. J. Fish Biol. 55, 158–171. 10.1111/j.1095-8649.1999.tb00665.x

[B54] Ye H. Huang H. Li S. Wang G. (2008). Immunorecognition of Estrogen and Androgen Receptors in the Brain and Thoracic Ganglion Mass of Mud Crab, Scylla Paramamosain. Prog. Nat. Sci. 18, 691–695. 10.1016/j.pnsc.2007.12.012

[B55] Zhou X. Song C. Grzymala T. L. Oi F. M. Scharf M. E. (2006). Juvenile Hormone and colony Conditions Differentially Influence Cytochrome P450 Gene Expression in the Termite Reticulitermes Flavipes*. Insect Mol. Biol. 15, 749–761. 10.1111/j.1365-2583.2006.00675.x 17201768

