# Supplementary material for: Recognition of Gonadal Development in Eriocheir sinensis Based on the Impulse of Love at First Sight
Source: Front Physiol. 2022 Apr 27;13:793699. doi: 10.3389/fphys.2022.793699 (PMC9091178; doi:10.3389/fphys.2022.793699)
Supplement: Supplementary file 1 [file DataSheet1.doc]

Table.S1 Verified Differentially Expressed Genes (DEGs)

| Gene | Sequence (5'-3') |
| --- | --- |
| GAPDH | F- GCGTGTTCACCACCATTGAG |
|  | R- ACATGGGTGCATCAGCAGAG |
| 18Srna | F- TCCAGTTCGCAGCTTCTTCTT |
|  | R- AACATCTAAGGGCATCACAGA |
| β-actin | F- GCATCCACGAGACCACTTACA |
|  | R- CTCCTGCTTGCTGATCCACATC |
| anti-lipopolysaccharide factor 3 | F- CAGAGAAGTAAAGGTAGGCGAGG |
|  | R- TTCATGTGTTACGGGTTGACGTT |
| Low-density lipoprotein receptor | F- GAACTCTCTGGGCTTAGTCTGG |
|  | R- AGACAGAGAATAGGGGACAGACA |
| cytochrome P450 49a1 | F- AGCGGTTGCACAAGTATTGGATA |
|  | R- GAGTCGCTTGAGTGTTAGGAGTG |
| alkaline phosphatase | F- TGGAATTCGAGAAGGCGATCAG |
|  | R- CTGGTAACCGTTGATCATGAGGG |
| cytochrome P450 CYP2B | F- ATGGCGTTGTCTAGTCTTAGTGG |
|  | R- ACTGAAGGCGAGATAAGAAGAGAG |
| Krueppel homolog 1 | F- CTTCCGTATTATTTGCCGTAACTC |
|  | R- CAAGAGTGTGGCAGAAGCTTTAT |
| juvenile hormone esterase | F- ATCACTGTAGCTTCTGCTCCTTG |
|  | R- TCGAAGTTGAAGAGAGAGAATGTG |
| C-type lectin | F- CCATGTTGGAATGACTGAGATG |
|  | R- TCCGAGGTATGGGTAGAAAATG |
